# Supplementary material for: Viruses under the Antarctic Ice Shelf are active and potentially involved in global nutrient cycles
Source: Nat Commun. 2023 Dec 14;14:8295. doi: 10.1038/s41467-023-44028-x (PMC10721903; doi:10.1038/s41467-023-44028-x)
Supplement: Supplementary file 1 — Supplementary Information [file 41467_2023_44028_MOESM1_ESM.pdf]

Supplementary Information for

Viruses under the Antarctic Ice Shelf are active and potentially involved in global nutrient cycles

Javier Lopez-Simon[1], Marina Vila-Nistal[1], Aleksandra Rosenova[1], Daniele De Corte[2,3], Federico Baltar[4]*, and Manuel Martinez-Garcia[1,5]*

[1]Department of Physiology, Genetics, and Microbiology, University of Alicante, Carretera San Vicente del Raspeig, San Vicente del Raspeig, Alicante, 03690, Spain

[2]Institute for Chemistry and Biology of the Marine Environment, Carl von Ossietzky University of Oldenburg, Oldenburg, Germany

[3]Ocean Technology and Engineering, National Oceanography Centre, Southampton, UK

[4]Department of Functional & Evolutionary Ecology, University of Vienna, Djerassi-Platz 1, 1030 Vienna, Austria

[5]Instituto Multidisciplinar para el Estudio del Medio Ramon Margalef, University of Alicante, San Vicente del Raspeig, Alicante, 03690, Spain

*Correspondence to: m.martinez@ua.es and federico.baltar@univie.ac.at

-This supplementary material contains four figures

-Notice that the Source Data file attached to this manuscript contains source data complementing and supporting our findings on virus features, genome sequence, predicted ORFs (protein sequence), viral classification, viral network analysis, virus-host assignment, data on abundance and activity of viruses, similarity with vSAG 37-F6 virus and AMG information.

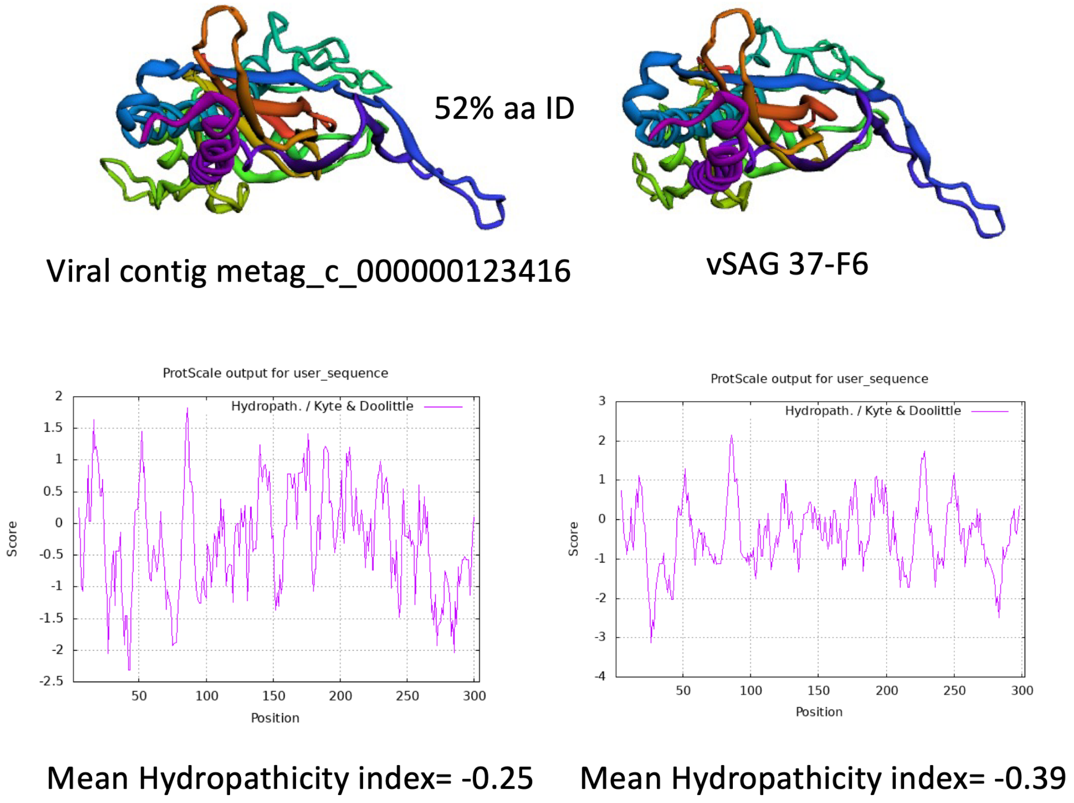

Figure S1. Genetic comparison and 3D structure prediction of capsid protein for some of the abundant viruses under the Ross Ice Shelf sharing orthologues hallmark genes with virus vSAG 37-F6. Amino acid similarity percentage is indicated for the product of ORF 9 encoding the capsid protein of virus vSAG 37-F6 also detected in viruses of the Ross Ice Shelf. 3D-structure protein prediction was made with Alphafold. Hydropathicity index for the capsid protein is indicated. See method for details.

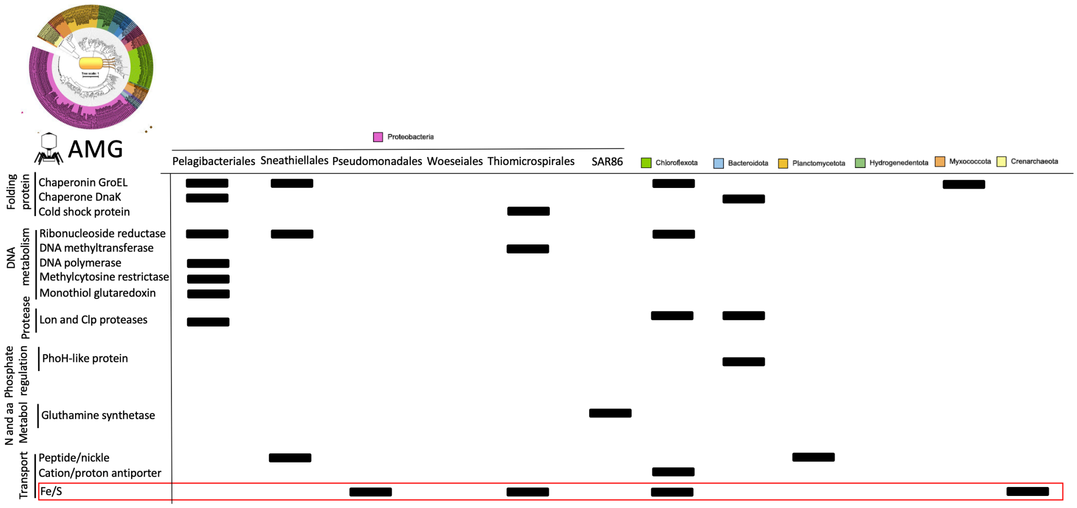

Figure S2. AMGs in viruses under the Ross Ice Shelf. Abundant viral AMG are depicted including the information of potential hosts. Some of the detected viral AMG belong to very abundant and active viruses that infect some of the most abundant and active chemolitoautotrophic bacteria contributing to primary production under the Ross Ice Shelf according to [18 see references in main text]. 3D-structure protein prediction was made for some AMG involved in sulfur transportation. Notice that the 3D structure of the homologue viral protein and that of the host are quite similar. Prediction was performed with Alphafold.

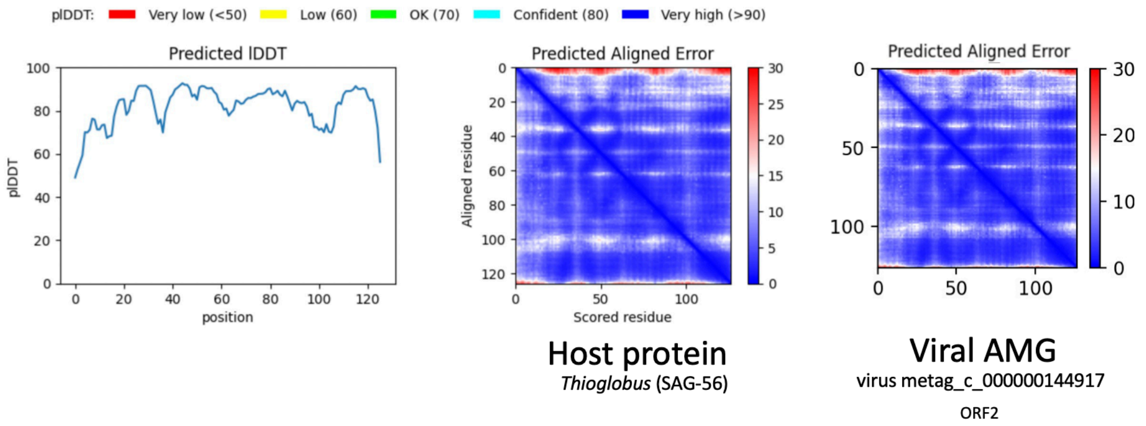

Figure S3. Confident values obtained from Alphafold for the folding prediction of AMG protein (ORF2) involved in S/Fe transportation present in virus contig "metag_c_000000144917" (and other viruses) and its corresponding homologue protein in *Thioglobus* spp. As shown, for most of the 90% of the protein length, the predicted aligned error was very low indicating that the prediction was highly confident. Similar results was obtained for the other 3D folding structure depicted in figure 4.

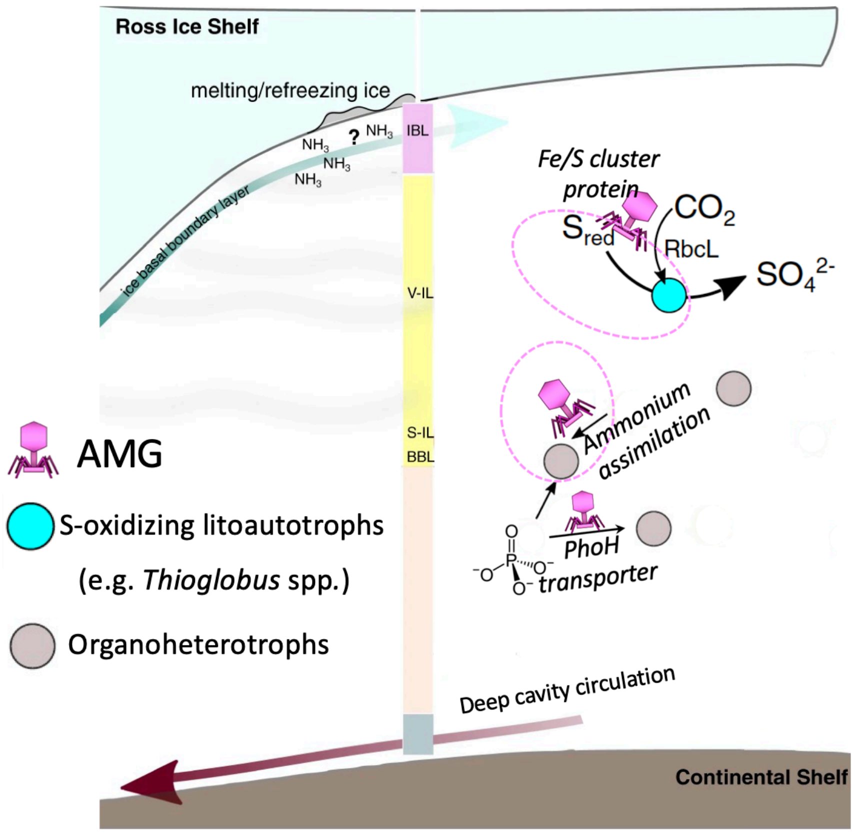

Figure S4. Conceptual diagram illustrating the contribution of viruses inhabiting under the Ross Ice Shelf to global carbon and nutrient cycles. At the basal part of the ice shelf, high concentration of ammonium was found (see paper by Martinez-Perez et al 2022), albeit its origin is uncertain. Key viral AMGs involved in nitrogen and ammonium assimilation, and other key nutrients, such as phosphorous were found. High abundance of viral AMGs involved potentially in sulfur transportation were detected in viruses infecting ecologically important prokaryotes in the RIS that sustain primary production. IBL, Ice basal boundary layer. V-IL, variable intermediate layer, S-IL, stratified intermediate layer. BBL, benthic boundary layer. This figure has been modified from Martinez-Perez et al 2022; https://doi.org/10.1038/s41467-021-27769-5. For more details about the structure and ocean cavity circulation under the RIS, please see that publication.
