# Supplementary material for: Viruses under the Antarctic Ice Shelf are active and potentially involved in global nutrient cycles
Source: Nat Commun. 2023 Dec 14;14:8295. doi: 10.1038/s41467-023-44028-x (PMC10721903; doi:10.1038/s41467-023-44028-x)
Supplement: Supplementary file 3 — Reporting Summary [file 41467_2023_44028_MOESM3_ESM.pdf]

nature portfolio

Corresponding author(s): Manuel Martinez-Garcia

Last updated by author(s): 2023/11/08

Reporting Summary

Nature Portfolio wishes to improve the reproducibility of the work that we publish. This form provides structure for consistency and transparency in reporting. For further information on Nature Portfolio policies, see our Editorial Policies and the Editorial Policy Checklist.

Statistics

For all statistical analyses, confirm that the following items are present in the figure legend, table legend, main text, or Methods section.

n/a	Confirmed	
☐	☒	The exact sample size (*n*) for each experimental group/condition, given as a discrete number and unit of measurement
☐	☒	A statement on whether measurements were taken from distinct samples or whether the same sample was measured repeatedly
☒	☐	The statistical test(s) used AND whether they are one- or two-sided<br>*Only common tests should be described solely by name; describe more complex techniques in the Methods section.*
☒	☐	A description of all covariates tested
☒	☐	A description of any assumptions or corrections, such as tests of normality and adjustment for multiple comparisons
☐	☒	A full description of the statistical parameters including central tendency (e.g. means) or other basic estimates (e.g. regression coefficient) AND variation (e.g. standard deviation) or associated estimates of uncertainty (e.g. confidence intervals)
☒	☐	For null hypothesis testing, the test statistic (e.g. *F*, *t*, *r*) with confidence intervals, effect sizes, degrees of freedom and *P* value noted<br>*Give P values as exact values whenever suitable.*
☒	☐	For Bayesian analysis, information on the choice of priors and Markov chain Monte Carlo settings
☒	☐	For hierarchical and complex designs, identification of the appropriate level for tests and full reporting of outcomes
☒	☐	Estimates of effect sizes (e.g. Cohen's *d*, Pearson's *r*), indicating how they were calculated

Our web collection on statistics for biologists contains articles on many of the points above.

Software and code

Policy information about availability of computer code

Data collection	EMBL Nucleotide Sequence Database (ENA) database under Bioproject PRJEB35712
Data analysis	Softwares and version used are: Virsorter version 2.0, CheckV v1.01, PPR-Meta v1.1, geNomad v1.7.1, Vcontact version2.0, NCBI BLAST ncbi-blast-2.15.0+, WIsH v1.1, Protscale Expasy tool (https://web.expasy.org/protscale/) ,

For manuscripts utilizing custom algorithms or software that are central to the research but not yet described in published literature, software must be made available to editors and reviewers. We strongly encourage code deposition in a community repository (e.g. GitHub). See the Nature Portfolio guidelines for submitting code & software for further information.

Data

Policy information about availability of data

All manuscripts must include a data availability statement. This statement should provide the following information, where applicable:

- Accession codes, unique identifiers, or web links for publicly available datasets
- A description of any restrictions on data availability
- For clinical datasets or third party data, please ensure that the statement adheres to our policy

The original sequenced metagenomic and metatranscriptomic raw and assembled data including contigs mined and identified here as viruses along with single-amplified genomes and metagenome-assembled genomes, were available at EMBL Nucleotide Sequence Database (ENA) database under Bioproject PRJEB35712. Data on viral contigs (fasta nucleotide and amino acid sequences) are also available in the Supplementary Dataset that support and complement the manuscript. This comprehensive Supplementary dataset additionally contain data on viral classification, viral network analysis, virus-host assignment, transcriptional activity of

viruses, and data on AMG information. The following public databases were used in this study: geNomad database https://zenodo.org/records/8339387. IMG_VR_2022-09-20_7.1 - IMG/VR v4.1 (viral database) https://genome.jgi.doe.gov/portal/IMG_VR/IMG_VR.home.html, NCBI.

Research involving human participants, their data, or biological material

Policy information about studies with human participants or human data. See also policy information about sex, gender (identity/presentation), and sexual orientation and race, ethnicity and racism.

Reporting on sex and gender	n/a
Reporting on race, ethnicity, or other socially relevant groupings	n/a
Population characteristics	n/a
Recruitment	n/a
Ethics oversight	n/a

Note that full information on the approval of the study protocol must also be provided in the manuscript.

Field-specific reporting

Please select the one below that is the best fit for your research. If you are not sure, read the appropriate sections before making your selection.

[x] Life sciences [ ] Behavioural & social sciences [ ] Ecological, evolutionary & environmental sciences

For a reference copy of the document with all sections, see nature.com/documents/nr-reporting-summary-flat.pdf

Life sciences study design

All studies must disclose on these points even when the disclosure is negative.

Sample size	We do not conduct sampling in this survey. Sampling and sample size is described previously in Martinez-Perez et al 2022 (Nat Comm)
Data exclusions	No data were excluded from the analysis
Replication	Replication for sequencing data was successful and described previously in Martinez-Perez et al 2022 (Nat Comm).
Randomization	Sampling in the Antarctic continent in the Ross Ice Shelf for performing a drilling campaign was not at random since it was selected based on logistic criteria set by a multidisciplinary and trans-continetal team of researchers. See location of sampling in a previous paper byMartinez-Perez et al 2022 (Nat Comm).
Blinding	Blinding sampling in the Antarctic continent in the Ross Ice Shelf for performing a drilling campaign does not make any sense because sampling and location is based on scientific, technical and logistic criteria set by a multidisciplinary and trans-continetal team of researchers. See location of sampling in a previous paper byMartinez-Perez et al 2022 (Nat Comm).

Reporting for specific materials, systems and methods

We require information from authors about some types of materials, experimental systems and methods used in many studies. Here, indicate whether each material, system or method listed is relevant to your study. If you are not sure if a list item applies to your research, read the appropriate section before selecting a response.

Materials & experimental systems

n/a	Involved in the study	
[x]	[ ]	Antibodies
[x]	[ ]	Eukaryotic cell lines
[x]	[ ]	Palaeontology and archaeology
[x]	[ ]	Animals and other organisms
[x]	[ ]	Clinical data
[x]	[ ]	Dual use research of concern
[x]	[ ]	Plants

Methods

n/a	Involved in the study	
[x]	[ ]	ChIP-seq
[x]	[ ]	Flow cytometry
[x]	[ ]	MRI-based neuroimaging
